# Antihyperglycemic and Antilipidemic Properties of a Tea Infusion of the Leaves from *Annona cherimola* Miller on Streptozocin-Induced Type 2 Diabetic Mice

**DOI:** 10.3390/molecules26092408

**Published:** 2021-04-21

**Authors:** Jesús Martínez-Solís, Fernando Calzada, Elizabeth Barbosa, Miguel Valdés

**Affiliations:** 1 Instituto Politécnico Nacional, Sección de Estudios de Posgrado e Investigación, Escuela Superior de Medicina, Plan de San Luis y Salvador Díaz Mirón S/N, Col. Casco de Santo Tomás, Miguel Hidalgo, Mexico City 11340, CP, Mexico; rbarbosa@ipn.mx (E.B.); valdesguevaramiguel@gmail.com (M.V.); 2 Unidad de Investigación Médica en Farmacología, UMAE Hospital de Especialidades 2° Piso CORSE Centro Médico Nacional Siglo XXI, Instituto Mexicano del Seguro Social, Av. Cuauhtémoc 330, Col. Doctores, Cuauhtémoc, Mexico City 06720, CP, Mexico

**Keywords:** *Annona cherimola* Miller, antihyperglycemic activity, antilipidemic activity, HPLC analysis, toxicity

## Abstract

The antihyperglycemic and antilipidemic effects of the tea infusion extracts of leaves from *Annona cherimola* Miller (IELAc-0.5, IELAc-1.5, and IELAc-3.0) were evaluated on normoglycemic (NG) and streptozocin-induced diabetic (STID) mice. In the acute test, IELAc-1.5 at 300 mg/kg bodyweight (bw) exhibited antihyperglycemic activity on STID mice since the first hour of treatment. Then, its antidiabetic potential was analyzed in a subchronic evaluation. IELAc-1.5 was able to reduce the blood glucose level, glycated hemoglobin (HbA1c), cholesterol (CHO), and triglycerides (TG); high-density lipoprotein (HDL) showed an increase at the end of treatment. IELAc-1.5 did not modify the urine profile at the end of the evaluation, and neither toxicity nor macroscopic organ damage were observed in acute and subchronic assays. In addition, a major flavonol glycoside present in the tea infusion extracts was identified using high-performance liquid chromatography with diode array detection (HPLC-DAD). The analysis of the tea infusion extracts by HPLC revealed that rutin was the major component. This study supports the use of tea infusions from *Annona cherimola* for the treatment of diabetes and suggests that rutin could be responsible, at least in part, for their antidiabetic properties.

## 1. Introduction

Diabetes mellitus (DM) is a multifactorial disease characterized by a chronic hyperglycemic state. This is a consequence of resistance to or lack of insulin secretion from the pancreas [1] as well as other carbohydrate and lipid disturbances, such as dyslipidemia; all of those factors are known collectively as the ominous octet [2], which lead to micro- and macrovascular complications [3]. DM type 2 is the most frequent type globally, accounting for 90–95% of all cases of diabetes, affecting over 463 million people [4], and causes almost 5 million deaths per year [5]. Currently, there are a wide range of antidiabetic drugs classified as secretagogues (sulfonylureas and meglitinides), sensitizers (metformin and thiazolidinediones), or antihyperglycemic drugs, including α-glucosidase, dipeptidyl peptidase-IV (DPP-IV), or sodium-glucose cotransporter 2 (SGLT2) inhibitors and GLP-1 analogues; all of these are used alone or in combinations [6]. These antidiabetic drugs exert effects to improve glucose uptake or avoid blood glucose level increases; however, their use for extended periods is limited due to the undesirable side effects caused, such as gastrointestinal disorders, increased body weight, and others of greater severity [7]. Thus, the search for alternative treatments is necessary to help control this disease.

On the other hand, tincture preparations or tablets and supplements based on herb extracts are available in pharmacies, with or without prescription, for the treatment of some chronic diseases, including diabetes and its associated complications [8]. Infusions (teas, tisanes) or decoctions with the powder of the leaves, flowers, seeds, and fruits from medicinal plants constitute the preferred mode of preparation by different traditional medicine practitioners in different parts of the world [9,10] because of their, antioxidant, hypoglycemic, hypolipidemic, and antihypertensive properties [11], and they can be used alone or as polyherbal preparations [12]. Although some of these herbal-based preparations have no undesirable side effects, further studies are necessary to validate their use as supplements for human consumption [13].

*Annona cherimola* Miller (*A. cherimola*) is an evergreen fruit tree belonging to the *Annonaceae* family [14], known as “annona” or “cherimoya” [15,16], and is distributed in subtropical areas around the world [17]. Local populations in México use their leaves to treat gastrointestinal disorders, worms, and diarrhea [18]. Similarly, extracts from other species of the genus *Annona* are also used for wide beneficial purposes [19,20,21]. Recently, studies have listed the compounds present in *A. cherimola* leaves [22] and highlighted their contents of flavonoids and other phenolic compounds, which could have a wide number of properties [23,24]. Studies have proven that alcoholic extracts obtained from the leaves of this species have antidepressant [25] and pro-apoptotic [26] activities. Taking into consideration the relevance of the treatment of diabetes and its complications with herbal medicine, we decided to analyze the antihyperglycemic and antilipidemic effects of tea infusion of the leaves from *A. cherimola*.

Thus, in order to support the use of these beverages as a new possible alternative medicine for diabetes treatment, in the present work, we assess the antihyperglycemic activity of different tea infusion extracts of the leaves from *A. cherimola* (IELAc-0.5, IELAc-1.5, and IELAc-3.0) in normoglycemic (NG) and streptozocin-induced diabetic (STID) mice. The tea infusion extract that exerted the best effect was further evaluated for antidiabetic and antilipidemic properties in a subchronic assay measuring the weight loss, blood glucose level, glycated hemoglobin (HbA1c), cholesterol (CHO), triglycerides (TG), and high-density lipoprotein (HDL); the urine profile was also evaluated. Additionally, the major flavonoid glycoside was determined using high-performance liquid chromatography with diode array detection (HPLC-DAD) analysis. At the end of the assay, a macroscopic examination was conducted and the relative organ weight (ROW) of the treated mice was assessed.

## 2. Results

### 2.1. Obtaining of the Tea Infusion Extracts of the Leaves from Annona cherimola

For the elaboration of the tea infusion extracts, amounts of 0.5, 1.5, and 3.0 g of leaves powder from *A. cherimola* were placed inside tea bags, each with the objective of choosing the amount which obtains the best yield of extract and rutin, which was used as an activity marker. As shown in Table 1, the highest amount of the flavonoid glycoside rutin was obtained with the tea infusion extract made with 1.5 g of plant powder (IELAc-1.5), while the yield of extract was higher in the tea infusion extract with 3.0 g plant powder (IELAc-3.0). As the main active flavonoid rutin in the extract was obtained in important concentrations with IELAc-1.5, this tea infusion was selected for the next step of research; however, the extraction procedure could have allowed the obtaining other components of the leaves that contributed to obtaining a high yield of the infusion extract IELAc-3.0.

### 2.2. In Vivo Acute Assay

#### 2.2.1. Acute Antihyperglycemic Activity of the Tea Infusion Extracts from *Annona cherimola* (IELAc-0.5, IELAc-1.5, and IELAc-3.0)

The acute antihyperglycemic activity was evaluated to determine the best antidiabetic potential of the three tea infusion extracts obtained using the leaves from *A. cherimola* (IELAc) made with 0.5 (IELAc-0.5), 1.5 (IELAc-1.5), and 3.0 g (IELAc-3.0) of plant powder. In this analysis, acarbose (Aca), an α-glucosidase inhibitor, was used as the reference drug as it has a similar mechanism of action and also several glycoside flavonoids, including rutin. After administration of a single oral dose of IELAc at 300 mg/kg bw and acarbose (50 mg/kg bw) to STID mice, their blood glucose levels were decreased significantly compared with the STID control mice, and there was no hypoglycemia in the treated animals in any case. A single administration of IELAc-1.5 and IELAc-3.0 exhibited a significant reduction in blood glucose level from 3 to 7 h post-administration (Table 2), causing a significant decrease of 55.8% compared to the STID mice and 62.9% compared to the initial values, whereas administration of the standard drug, acarbose, caused an antihyperglycemic effect at 1 to 5 h post-administration and raised to hyperglycemic values at 5 h post-treatment. IELAc-0.5 administration showed antihyperglycemic effects at 1 to 3 h post-administration in STID mice.

#### 2.2.2. Safety of a Single Dosage of the Tea Infusion Extract of Leaves from *Annona cherimola*

As the highest amount of rutin and the best acute antihyperglycemic effect were obtained with IELAc-1.5, it was used to evaluate the safety for further studies. To evaluate the safety of single, high doses of IELAc-1.5 in mice, the tea infusion extract was administrated to healthy mice at 30, 300, and 3000 mg/kg bw once, which allowed for estimating its Lethal Dose 50 (LD_50_) in accordance with the Organization for Economic Co-operation and Development (OECD) guideline No. 423 [27,28]. We analyzed whether the dose given caused lethality, behavior alterations, or macroscopic injury and weight loss. Treatment with the highest dose of IELAc-1.5 in mice did not cause changes in behavior or signs of neurological toxicity and did not modify the natural stool and urine during the period of 14 days of observation. Moreover, no mortality was observed in animals treated at all dose levels from the critical 24 h post-administration to the end of the first week. After sacrifice on the 14th day, macroscopic pathology observation of the internal organs no revealed visible lesions on any animal. Moreover, there were no significant changes or macroscopic tissue injury or loss of weight of the internal organs compared with the control group. Therefore, according to the chemical labeling and classification of acute systemic toxicity recommended by the Organization for Economic Co-operation and Development (OECD, 2001), the following categories were determined: category 1, very toxic ≤ 5 mg/kg; category 2, toxic > 5 and ≤ 50 mg/kg; category 3, harmful > 50 and ≤ 300 mg/kg; category 4, low risk > 300 and ≤ 2000 mg/kg; and category 5, safe or no label > 2000 mg/kg. The theoretical LD_50_ value of IELAc-1.5 is greater than 3000 mg/kg, thus suggesting that it is safe to use in humans.

### 2.3. Subcrhonic Assay

#### 2.3.1. Effect of Continued Administration of the Tea Infusion Extract of Leaves from *Annona cherimola* on Blood Glucose Levels Measurement in the Subchronic Assay

In the subchronic assay, IELAc-1.5 was administered to healthy and STID mice for 28 days. To evaluate the antidiabetic potential of the tea infusion extract of the leaves from *Annona cherimola* (IELAc-1.5), the blood glucose level was measured weekly. In the STID control group, there was a significant gradual increase in blood glucose level compared with the initial values. Similarly, a gradual increase was observed in the group treated with acarbose at the end of the assay. Interestingly, the administration of IELAc-1.5 exhibited control of blood glucose levels in the STID mice from 21 days to the end of the treatment (Table 3). The NG mice treated with vehicle and IELAc-1.5 showed normal blood glucose levels throughout the entire treatment.

#### 2.3.2. Effect of the Tea Infusion Extract of Leaves from *Annona cherimola* (IELAc-1.5) on Lipid Profile and Glycated Hemoglobin (HbA1c)

To evaluate the antilipidemic and glycated hemoglobin (HbA1c) reduction properties after continuous administration of IELAc-1.5, biochemical analyses of glycated hemoglobin (HbA1c), cholesterol (CHO), triglycerides (TG), high-density lipoprotein (HDL), and low-density lipoprotein (LDL) levels were performed. The STID control mice showed a significant increase in the level of HbA1c compared with NG mice. It should be noted that the administration of IELAc-1.5 for 28 days to STID mice significantly reduced the HbA1c value with respect the acarbose and STID control groups (Table 4). On the other hand, the NG mice treated with IELAc-1.5 did not show differences in lipid profile throughout the treatment period. Additionally, the administration of IELAc-1.5 caused a significant decrease in cholesterol and triglycerides at the end of the treatment with respect to the STID control mice to almost normal values. Moreover, the administration of IELAc-1.5 in STID mice increased the HDL value at the end of the treatment compared with NG mice and decreased the LDL value with respect to the STID control mice.

#### 2.3.3. Effect of the Administration of the Tea Infusion Extract of Leaves from *Annona cherimola* (IELAc-1.5) on Weight Lost in Subchronic Assay

**Figure 1:**
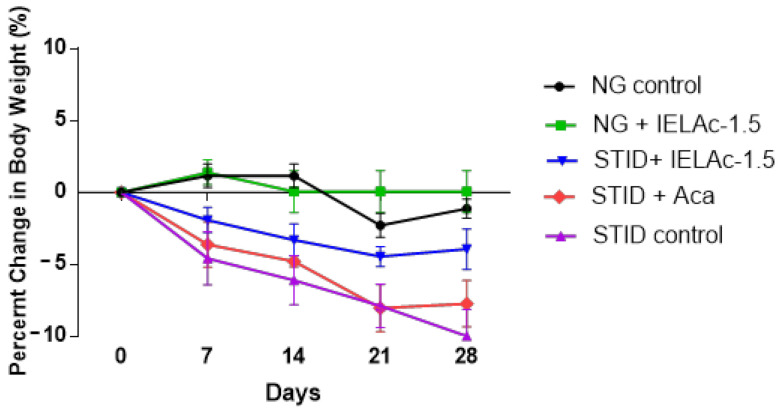
Mean percentage (%) of change in body weight (bw) of experimental groups in subchronic assay. Each point represents the mean value of each group (*n* = 6). All measurements of % change in bw were compared with the bw at beginning of the assay. NG, normoglycemic; STID, streptozocin-induced diabetic mice; IELAc-1.5, group administered tea infusion extract with 1.5 g of the leaves from *A. cherimola* 300 mg/kg; Aca, group administered acarbose 50 mg/kg as pharmacological control.

The IELAc-1.5 administered for 28 days to NG mice did not cause changes in behavior during the treatment. Additionally, there was no significant difference in the weekly body weight of normoglycemic (NG) treated mice between the beginning and end of treatment (Table 5). Moreover, no death was recorded after daily administration of treatment for four weeks in either the control or the extract group. Furthermore, no differences were observed in the percentage (%) of body weight in both NG groups. However, there was a significant gradual decrease in % of body weight in the STID control and acarbose-treated groups. Besides, the IELAc-1.5-treated group showed a significant reduction in body weight loss at the end of the treatment compared with all of the other STID groups. The changes in the percentage (%) of body weight were measured by comparing the value at the beginning of the assay with that at the end (Figure 1).

#### 2.3.4. Effect of the Tea Infusion Extract of *Annona cherimola* on Stool and Urine Profile on Subchronic Assay

Weekly evaluations of treated NG and STID mice did not show changes in natural stool at the end of treatment, while an increase in the urine glucose value was observed in the STID control group. The IELAc-1.5-treated STID group showed a significant decrease in urinary glucose concentration in comparison with the vehicle and acarbose groups (Table 6). Other parameters had not changed in all cases.

#### 2.3.5. Effects of the Tea Infusion Extract of Leaves from *Annona cherimola* (IELAc-1.5) on Weight of Internal Organs in Treated Mice

To investigate the effect of IELAc-1.5 on the changes in the organ weight index in the diabetic condition, the weights of the pancreas, liver, spleen, kidneys, gut, and stomach were measured in the NG and STID mice and calculated as an index by the ratio (%) of organ weight per bw. The analysis of the internal organs on NG mice treated with IELAc-1.5 and vehicle did not show differences in relative weights (Table 7) or in the macroscopic examination. In addition, the macroscopic examination did not show any changes in the color of the organs of the treated animals compared with vehicle-treated animals. Treatment with IELAc-1.5 at 300 mg/kg appeared to not affect healthy visceral organs in NG mice. In the case of the STID groups, there were changes in the surface and relative weight of the kidneys. The relative weight of the pancreas in the STID control group was lower compared to the NG groups. Additionally, pallor was observed on the pancreatic surface in these groups.

### 2.4. HPLC Analysis of the Tea Infusion Extracts

**Figure 2:**
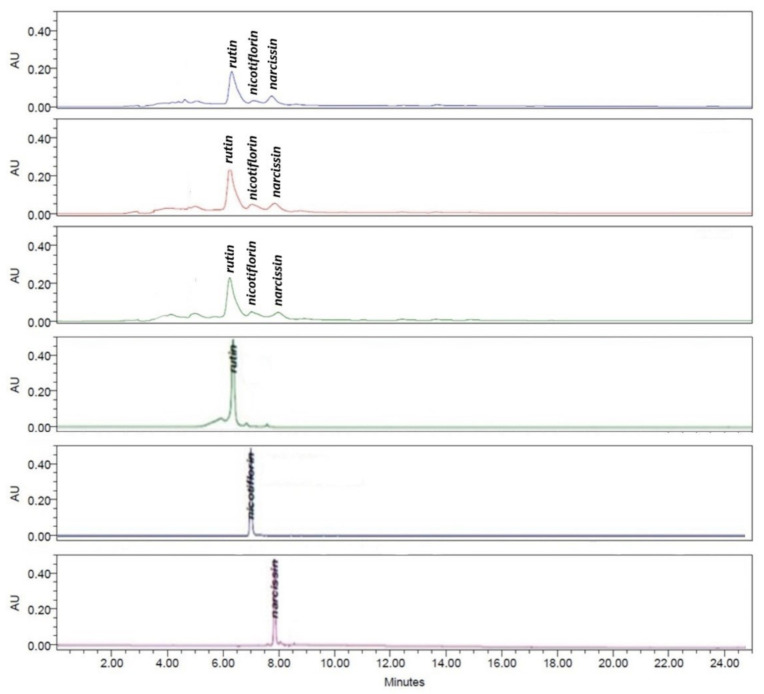
High-performance liquid chromatography with diode array detection (HPLC-DAD) analysis at 254 nm of tea infusion extracts of the leaves from *A. cherimola*: IELAc-0.5 (blue), IELAc-1.5 (red), IELAc-3.0 (green), and the flavonoid standards rutin, nicotiflorin and narcissin.

Analysis of the tea infusion extracts IELAc-0.5, IELAc-1.5, and IELAc-3.0 was performed using high-performance liquid chromatography with diode array detection (HPLC-DAD) and standards of the flavonol glycosides rutin, nicotiflorin, and narcissin were used. The analysis showed the presence of high levels of rutin at 6.245 min, and minor concentrations of nicotiflorin at 7.197 min and narcissin at 7.752 min in IELAc-0.5, IELAc-1.5, and IELAc-3.0 (Figure 2). The identification was made by comparing their retention times, ultraviolet spectra, and thin layer chromatography.

## 3. Discussion

Diabetes mellitus management is an important public health problem that needs new alternatives. Although many attempts have been made to treat the disease, the objectives have not yet been achieved due to several limitations of the current therapies [3]. This leads to the need to find complementary treatments that are effective and without undesirable side effects [29]. One of the main sources of treatment is medicinal plants that have been used in traditional medicine for different purposes [30]. In this sense, there are studies that show species known for their antidiabetic properties [31,32] and that are used as supplements to help achieve glycemic control in patients with diabetes [33].

Furthermore, medicinal plants of the genus *Annona* have been studied due to their properties for treating several illnesses [19,20,21]. Several studies using aqueous and alcoholic extracts of *Annona muricata*, *A. squamosa, A. stenophylla*, *A. macroprophyllata*, and *A. diversifolia* have already shown their antidiabetic, antioxidant, antilipidemic, and antinociceptive effects, which are mainly attributed to the presence of phenolic compounds in their leaves [34,35,36,37,38,39,40,41,42,43,44]; therefore, they are a good source to search for new compounds with medicinal properties.

The present study resulted from the increasing interest on the use of preparations and beverages made with medicinal plants and commonly used in herbal medicine and the need to provide scientific evidence that the traditional medicinal use of *A. cherimola* could be justified. Additionally, it was conducted in order to develop a new alternative treatment for diabetic patients’ consumption.

Although there have been studies on the effect of *A. cherimola* aqueous extract, there are not enough to show the effect of tea infusion of this species on blood glucose levels. To date, *A. cherimola* alcoholic extract has been considered a promising natural remedy for hyperlipidemia, which may contribute to exert anti-atherosclerotic activity [39]. Moreover, recent studies [45] have demonstrated that leaf decoctions inhibited the 3-hydroxy-3-methyl-glutaryl-CoA (HMG-CoA) reductase activity and decreased the cholesterol uptake in intestinal cells. This effect is attributed to the presence of flavonoids in the decoction. Additionally, the known healthy phenolic compounds in this species could prevent or delay disorders caused by oxidative derangements [46], and it is known that the leaves are a good source to develop dietary supplements to manage redox balance [24]. However, more studies that support the use of infusions for diabetes treatment in human beings are still necessary. Based on this, we decided to assess the effect of treatment with the infusions of the leaves from *A. cherimola* on the main blood parameters related with the diabetic condition and to evaluate the security as well as changes to visceral organs by using an STID mouse model.

Phytochemical analysis helps to detect the chemical constituents of plant extracts to find the major active compound as a basis for drug development [47]. In this work, the analysis of the tea infusion extract of the leaves from *A. cherimola* showed flavonol glycosides to be the main components, which are related to its pharmacological effect. The extraction procedure can increase the amount of phenolic compounds in the aqueous extracts, including flavonoids, using the Maillard reaction, which occurs as the result of increasing the temperature of the aqueous solution [48]. Furthermore, leaf extracts obtained under subcritical fluid conditions at a high temperature have a higher α-glycosidase inhibitory capability [49]. Moreover, the presence of flavonoids such as rutin in aqueous extracts is high, as compared with other compounds in the leaf extracts, and can contribute to the antioxidant [24] and antilipidemic [39] activities of *A. cherimola.* Our results confirm that infusion could be a good method to obtain rutin as the main active compound, with the amount being higher when beverages are made with 1.5 g of plant powder. Considering that the traditional way in which several species of this genus are consumed is as a decoction or infusion in boiled water [39], these results scientifically support that the infusion made with 1.5 g of leaves from *A. cherimola*, similar to its common use, is a correct procedure to achieve an expected effect.

Previous studies indicated an antihyperglycemic effect on the polar fraction of this species [50], which was similarly found for other *Annona* species, occurring in an aqueous extract of *A. squamosa* [37]. In our study, the assessment of the infusions showed a decrease in the plasma glucose level at 300 mg/kg compared with the vehicle group in the acute as well as the subchronic assay. Furthermore, during diabetes mellitus, the excess of glucose present in the bloodstream leads to an increase in free radicals, which react, by non-enzymatic oxidation, with hemoglobin to form HbA1c [51]. HbA1c reflects the average blood glucose level [52], and for a long time, it has been the gold standard of continuous monitoring as well as diagnostic criteria in patients with diabetes mellitus [53], since the amount of increase is proportional to the fasting blood glucose level. Our results show that continuous administration of the infusion extract in diabetic mice causes a decrease in the level of HbA1c following subchronic treatment for 28 days. That may reflect the extract-induced formation of advanced glycation end-products (AGEs). The presence of rutin as well as other related flavonoids in aqueous extracts could exert an antioxidant effect, which is related with their glucose-lowering diffusion properties [54]. Furthermore, this effect could be enhanced by their structure, which gives them high α-glycosidase and α-amylase inhibitory activity [55]. In both cases, the resultant effect is a decrease in the postprandial glucose rise and avoidance of the oxidative reaction as a result of high blood glucose levels. As previously shown, the analysis by HPLC-DAD was able to identify rutin in IELAc. Rutin is a glycoside derivate from quercetin, and both have great α-glucosidase activity and powerful antioxidant effects [56,57] as well as other activities [58], whose mechanisms are already known [59]. This flavonoid is also able to prevent streptozocin-induced oxidative stress and protect B cells from peroxidation, which increases insulin secretion and reduces the fasting blood glucose levels, finally leading to reduced formation of glycated hemoglobin [60]. As such, more than one mechanism may be involved in the response found [61]. Furthermore, these results are in accordance with the findings of recent studies [35] showing that phenolic compounds present in the polar fraction of *Annona muricata* have similar properties. The antihyperglycemic effect shown could be due to not only the flavonoid rutin but also other phenolic compounds in the leaves of this species [22,23], whose presence may be favored by the extraction process [48,49]. Additionally, according to these data about the antihyperglycemic effect on acute and subchronic evaluations as well as the lowering effect on glycated hemoglobin levels, the tea infusion extract of *A. cherimola* could be efficient as a long-term treatment. In contrast, acarbose, an α-glucosidase inhibitor used for the diabetes treatment exhibited a less effect in the acute and subchronic assay at 50 mg/kg, this effect is similar to was found in other previous studies with the same dose [62], and are consistent with studies where the administration of acarbose alone generated less control over the hyperglycemic state of STZ-induced diabetic animals, while treatment with flavonoids such as quercetin improves not only the postprandial glucose rise but also fasting hyperglycemia [63], since it increases insulin sensitivity by inhibiting the α-glucosidase pathway and favoring the release of insulin from the pancreas by protecting the b cells from oxidative damage [56,57,58,59,60]. On the other hand, although acarbose is a powerful competitive inhibitor of α-glucosidase, it has been reported that its use for a long time induces side effects that limit its effectiveness. In addition, it has been observed that the plateau effect generated with doses at 50 mg and 100 mg of acarbose could be explained because during the chronic state of diabetes mellitus type 2, there are differences in the enzymatic activity of α-glucosidase, which leads to an overlapping of dose-responses. Furthermore, the effect on postprandial carbohydrate absorption exerted by acarbose could be limited due to a decrease in the ability to suppress endogenous glucose production [64]. Besides, due to the low absorption of acarbose into the bloodstream, could be an accumulation in the intestinal lumen which may cause saturation of the activity of the enzyme α-glucosidase, and result in a decrease in its long-term effectiveness.

Moreover, chronic hyperglycemia in diabetic people is related with increased cardiovascular risk due to oxidative stress, vascular damage [65], and dyslipidemia characterized by hypertriglyceridemia as well as high levels of cholesterol—mainly low-density lipoprotein (LDL) and low levels of high-density lipoprotein (HDL) [66,67]—and leads to an increased mortality risk associated with cardiovascular diseases and poorer prognosis compared to non-diabetic individuals [68]. Thus, the search for alternative treatments that could improve the lipid profile and modulating vascular changes associated with diabetes mellitus has received clinical interest to develop new treatments [69]. In our case, subchronic treatment with the infusion from *A. cherimola* was able to reduce the cholesterol and triglycerides levels in diabetic mice and led an increase in HDL at the end of the treatment. This effect is in concordance with a related published work [45], which showed lowered cholesterol absorption on a cell culture attributed to the presence of antilipidemic phenolic compounds in the extract. Additionally, previous reports indicated that high polar extracts from other species of the genus *Annona* have antilipidemic effects [34,36,70]. Moreover, the antioxidant and anti-inflammatory effects of flavonoids can also reduce hepatic lipid peroxidation and lead to a decrease in the synthesis of cholesterol [71,72]. The present results show that the infusion is able to induce a reduction in the triglycerides level, which could be influenced by the anti-peroxidation potential of the flavonoids in the extract. These data are interesting as they show that infusions from *A. cherimola* could have other therapeutic potential as they protect organs from inflammatory stress and atherosclerotic diseases, which lead to micro- and macrovascular damage associated with high blood glucose levels. They also agree with all other reports that show the beneficial effect of this species for several diabetic complications in preclinical and clinical assays [73]. Moreover, it is noteworthy that these data reflect that the use of an infusion of *A. cherimola*, as occurs in traditional medicine, could have a protective effect, and it is an indication that it is, perhaps, able to reduce the cardiovascular risk factors which contribute to the death of diabetic patients. The data provide further support for the traditional use of herbal formulations of *A. cherimola* as an agent not only to treat hyperglycemia but also to prevent several vascular complications related with diabetes mellitus.

In countries where people commonly use alternative therapeutics, medicinal plants are consumed in tea bags containing 2 g of plant powder per bag so that a 70-kg adult takes 0.11 g of plant material/kg/day [44]. Furthermore, beverages or tisanes (herbal teas) made from natural sources such as fruits and plants have high amounts of flavonoids, which are commonly consumed as aglycones or glycosides, from 650 mg to 1 g per day on average, and some dietary supplements based on quercetin have been marketed with a wide therapeutic range [74]. Our study showed that infusions from *A. cherimola* have flavonoid glycosides as the main components, and we decided to perform a toxicity study of this extract in accordance with the OECD guidelines [28]. Oral administration of the tea infusion extract did not cause changes in behavior or neurological toxic effects (dizziness, lethargy, or aggressiveness) in the animals treated with 300 and 3000 mg/kg. Based on this, the median acute toxicity value (LD_50_) of IELAc was estimated to be greater than 3 g/kg. This finding is similar with the report [47] showing that *Annona muricata* aqueous extract has a high LD_50_ value that indicates its safety for internal and external use in human beings. Therefore, the use of this infusion is safe for oral administration in relation to its traditional therapeutic use, since the theoretical LD_50_ is higher than what people usually consume by the traditional way.

Finally, body weight changes serve as a sensitive indication of the general health status of animals [46] and can also help in monitoring the efficacy of a treatment in diabetes mellitus. Continuous administration of the tea infusion extract did not cause any change in body weight in any group in the acute or subchronic evaluation at all doses used. Previous reports also indicates that analysis of glucose in urine of mice treated with the same vehicle showed a physiological urinary glucose excretion of almost 20 mg/dL [43]. The administration of IELAc-1.5 to NG mice did not modify the physiological values of the urine profile compared with the vehicle group, but reduces the urine glucose excretion in STID mice compared with other STID control groups. Also, reduced the body weight loss related with the subchronic condition in diabetic mice. It can be stated that the tea infusion extract from *A. cherimola* did not interfere with the normal metabolism of animals as demonstrated by the non-significant difference from normoglycemic animals in the vehicle control group. Furthermore, the macroscopic examinations of the organs of animals treated with this tea infusion extract did not show any changes in color compared with the vehicle control group. The difference between the relative weights of the pancreas in the treated groups is a sign of a possible protective effect of the tea infusion extract in these organs. The absence of organ toxicity in the normoglycemic mice confirms the safety of *A. cherimola* treatment. Our results show that there is a wide margin on the effective dose and that there is no toxic effect even at high dosage; therefore, it might be used as a rough indication of outcomes with human use. However, to establish supplement use, it is necessary to evaluate the effect of this extract during pregnancy; thus, further studies are being carried out.

## 4. Materials and Methods

### 4.1. General Information

Streptozocin (≥75% α-anomer basis, PN: S0130-5G), nicotinamide (≥99.5%, PN: 47865-U), and acarbose (PN: PHR1253-500MG) were purchased from Sigma-Aldrich^®^ (Sigma^®^, Saint Louis, MO, USA). Buffer solution (citric acid/sodium hydroxide/hydrogen chloride, pH 4.00, CC: 109445), saline solution 0.9% (solution 1000 mL), and DX-5 glucose solution 5% (solution 500 mL) were purchased from PISA^®^ Pharmaceutics (PISA^®^, Mexico City, México).

### 4.2. Plant Material

The leaves of *Annona cherimola* Miller were collected from a specimen with organic conditions in August 2019 in San Gregorio Atlapulco, Xochimilco, Mexico (19°15′08.3″ N 99°03′14.3″ W). The plant material was authenticated by the in-house botanist M.Sc. Santiago Xolapa of the Medicinal Plant Herbarium (IMSSM) in the Mexican Institute of Social Security (IMSS), where the voucher specimen is conserved under the reference number 16651. The name of the plant was verified at http://www.theplantlist.org (http://www.theplantlist.org/tpl1.1/record/kew-2640812). The leaves were washed twice with purified fresh water and left to dry in the dark at room temperature; later, they were finely powdered using a commercial blender and stored in air-tight Ziplock bags for further analysis.

### 4.3. Plant Extract Procedure

The extract of *A. cherimola* was made as a tea infusion, soaking a tea bag containing 0,5, 1.5 or 3.0 g of the leaves of plant material into 125 mL of boiled distilled water for 20 min to emulate the traditional use. The tea infusion extract (IELAc) obtained was then filtered through a grade 1 filter paper and concentrated by a rotary-evaporator (Büchi Labortechnik AG, Flawil, Switzerland) at 40 °C under reduced pressure. The yield of extraction was 563.69 mg of infusion extract/1.5 g of plant powder. The samples were analyzed by a HPLC-DAD (Waters Agilent, 5301 Stevens Creek Blvd Santa Clara, CA 95051, USA) to identify phenolic compounds into the extract. The sticky precipitate was stored at 4 °C until use. Aliquot of extract residue were weighed and re-suspended in water to be use.

### 4.4. Characterization of Flavonol on the Tea Infusion Extract of Leaves from A. Cherimola by High- Performance Liquid Chromatography

The IELAc was analyzed using HPLC–diode array detection (DAD) (Waters Agilent, 5301 Stevens Creek Blvd Santa Clara, CA 95051, USA). The analysis was performed using an HPLC-DAD Waters 2795 liquid chromatograph system coupled with a Waters 996 photodiode array detector and an analytical Millennium 3.1 workstation equipped with a C18 analytical column (Waters) with dimensions of 250 mm × 4.6 mm and particle size of 5 μm (Spherisorb S50D52, Waters Corporation, Milford, MA, USA)). For the analysis, 10mg of the tea infusion extract of leaves from *A. cherimola* (IELAc) was solved on 10 mL of ethanol, and a sample volume of 20 μL from that solution was injected. For elution, a system comprising a binary mobile phase of acetonitrile solvent/acetic acid 2% in water (A) and acetonitrile 100% (B) was used. The chromatograph operating conditions were programmed to give the following gradients: 1st stage—linear gradient of 80 (A)/20 (B) for 8 min; 2nd stage—linear gradient of 40/60 for 5 min; 3rd stage—linear gradient of 30/70 for 6 min; 4th stage—linear gradient of 90/10 for 6 min with a flow rate of 1 mL/min of mobile phase. The detection was made at a wavelength (λ) of 254 nm at room temperature and a total elution time of 25 min; at the end, the data collected were plotted. Reference standards of rutin as well as acetonitrile, ethanol, and acetic acid of HPLC grade were acquired from Sigma and were prepared and analyzed separately under the same conditions describe above. In all cases, the water used was of HPLC quality, purified in a Milli-Q system (Millipore, Bedford, MA, USA). The presence of substances in the tea infusion extract of leaves from *A. cherimola* was confirmed by comparing the retention times with the standard

### 4.5. Animals

Healthy male or female albino BALB/c mice aged 8–10 weeks (25 ± 5 g) were used in this study and obtained from the Animal House of the National Medical Center “Siglo XXI” from Instituto Mexicano del Seguro Social (IMSS). Investigations using experimental animals were conducted in accordance with the Official Mexican Regulations on animal care and experimental management [75]. The animals were housed under standard laboratory conditions at a temperature of 22 °C ± 2 °C with a 12-h light/dark cycle. Rodents were fed with a standard diet and purified water ad libitum. These studies were conducted with the approval of the Specialty Hospital Ethical Committee of the National Medical Center “Siglo XXI” from IMSS under register number R-2020-3601-038.

#### Grouping

Animals were assigned randomly to different groups of six mice each. Groups 1 and 2 were assigned as normoglycemic (NG) and streptozocin-induced diabetic (SITD) control mice, respectively, and treated with Tween 80% at 2% in distilled water. Group 3 (NG mice) and Group 4 (STID mice) received the tea infusion extract of leaves from *A. cherimola* (IELAc) at 300 mg/kg body weight of the rodent (bw). Group 5 of STID mice received 50 mg/kg bw of acarbose, which was used as a reference drug due to its mechanism of action as an α-glucosidase inhibitor being similar to that reported for flavonoid glycosides (Glucobay, tablets of 50 mg, Bayer). Blood samples were collected by puncture from the tail vein during the treatment. The glycemic value in each sample was assessed using the method described below. All treatments were administrated by the oral route through a gavage. The volume was calculated according to the OECD guidelines [27] as 2 mL/100 g bw.

### 4.6. Induction of Experimental Type 2 Diabetes

The animals were fasted overnight and experimental type 2 diabetes was induced by intraperitoneal injection of freshly prepared STZ (100 mg/kg bw) in 0.1 M citrate buffer (pH 4.5). After 30 min, a single intraperitoneal dose of nicotinamide (240 mg/kg) in saline solution 0.9% was given. The animals were allowed to drink 5% glucose solution overnight to stabilize the drug-induced hyperglycemia [37]. Blood glucose levels were determined after the last administration using the glucose oxidase method (Accu-Chek^®^ Performa Glucometer, Boehringer Mannheim, Germany) [76]. To confirm the STID model, a single dose of 5 mg/kg glibenclamide, a secretagogue drug was given orally to STZ-induced diabetic mice in order to confirm that the endocrine function of the pancreas is preserved and not completely obliterate due to the action of streptozocin [77]. Then, the animals with blood glucose values above 290 mg/dL and below 390 mg/dL on the third day after the STZ injection were considered as diabetic and used in this study.

### 4.7. Acute Effect and Toxicity Study

To evaluate the safety of a single dose of *A. cherimola* aqueous extract (AqEAc), the test guidelines (no. 423) on acute oral toxicity testing from OECD (2001) [27] were used. BALB/c mice that were fasted overnight but allowed free access to water ad libitum were randomly assigned into the following groups of three mice of either sex (three males or three females). The healthy control group received distilled water and the other three groups received a single dose of the AqEAc at 30, 300, and 3000 mg/kg bw respectively. The mice were not fed for 4 h following administration. Signs of toxic effects and neurotoxic signs such as dizziness, lethargy, and aggressiveness or mortality were observed 4 h after administration and then for the next 24 h. The general behavior of mice was observed daily for a period of 14 days for mortality, toxic effects, and/or changes in behavioral pattern. At the end of the experiments, the animals were euthanized according to NOM-062-ZOO-1999 [75]. Then, the internal organs (stomach, gut, kidneys, spleen, and liver) were extracted, weighted, and macroscopic observations were performed.

In the case of the acute effect study, STZ-induced diabetic mice were distributed as shown above. Treatments were given by the oral route and blood samples were collected by puncture from the tail vein at intervals of 0, 1, 3, 5, and 7 h after treatment. The glycemic value in each sample was assessed using a glucometer according to the method described above.

### 4.8. Subchronic Effect and Toxicity Study

This study was conducted to examine the long-term effects of daily oral administration of a tea infusion extract of leaves from *A. cherimola* (IELAc) on healthy and STZ-induced diabetic mice and was performed based on the OECD test guidelines 407 (2008) [28,78]. BALB/c mice were used and randomly divided into groups of 6 mice each, as was described previously. In this case, experimental diabetic groups were kept for 7 days before the beginning of the treatment to stabilize the diabetic condition of chronic hyperglycemia [50,79]. The animals received treatment orally by gavage for 28 consecutive days. All animals had free access to food and water throughout the duration of the experiment. At the end of the study, glycemia was measured in 40 μL of blood using a glucometer (Accu-Chek^®^ Performa Glucometer, Boehringer, Mannheim, Germany); glycated hemoglobin (HbA1c) was measured using a blood analyzer (Clover A1c Analyzer (Infopia Co., Ltd., Anyand, Korea) while total cholesterol, triglycerides, and high-density lipoprotein (HDL) levels were measured with 40 μL of blood samples obtained by puncture of caudal vain using the lipid panel of Cardiocheck Professional PA SILVER (Polymer Technology Systems, Whitestown, IN, USA), whereas low-density lipoprotein (LDL) values were calculated using Friedewald’s formula [80]. Animals were weighed on the first day (D0) and then weekly until the end of the assay. The percent change in body weight was calculated using the following formula (1):

Percentage change in body weight = 100 × {[Weight *n* − Weight *t*0]/Weight *t*0} (1)

where: Weight *t*0: measurement on the first day (D0); Weight *n*: weight measurements weekly.

At the end of the experiment, the animals were euthanized after treatment according to NOM0062-ZOO-1999 [67]. The internal organs were assessed by macroscopic analysis, as described above. The Organs of the sacrificed animals, including the spleen, pancreas, stomach, gut, liver, and kidneys, were excised, washed with saline solution 0.9%, weighed to obtain the absolute organ weight (AOW), and observed macroscopically. The relative organ weights (ROWs) were calculated for each animal using the following formula (2):

Relative Organ Weight (ROW) = {[AOW/Body weight at sacrifice]} × 100(2)

### 4.9. Statistical Analysis

Data are expressed as the mean ± standard error of the mean (SEM). Statistical significance was determined using a one-way analysis of variance (ANOVA) followed by the Bonferroni test for multiple mean comparisons (GraphPad Prism Version 6.01; GraphPad Software Inc., La Jolla, CA, USA), considering a *p*-value of < 0.05 as indicative of a significant difference between group means.

## 5. Conclusions

In this study, the administration of a single and repeated dose of the tea infusion extract of leaves from *Annona cherimola* Miller made with 1.5 g of plant powder (IELAc-1.5) and given at 300 mg/kg in STID mice improved the blood glucose level and glycated hemoglobin with control of lost body weight; it also had a beneficial effect on the level of cholesterol and triglycerides and increased the high-density lipoproteins. According to the OECD guidelines as well as the analysis of visceral organs, have high LD_50_. These results support that the use of tea infusions of leaves from *Annona cherimola* Miller could be a safe supplement for the treatment of diabetes mellitus type 2.

## Figures and Tables

**Table 1:** Yield of the extraction procedure and concentration of rutin into the three tea infusion extracts of leaves from *Annona cherimola* Miller.

Sample	Amount of Powder	Yield of Infusion Extract % (*w*/*w*, mg)	Rutin (mg/g of Tea- Infusion Extract)
IELAc-0.5	0.5 g	42.4 (212.2)	16.8
IELAc-1.5	1.5 g	36.7 (549.9)	23.6
IELAc-3.0	3.0 g	44.5 (1335.6)	20.0

**Table 2:** Effect of a single dose of the tea infusion extracts of *A. cherimola* on blood glucose levels of STZ-induced diabetic mice.

Group	Blood Glucose Levels (mg/dL)				
	0 h	1 h	3 h	5 h	7 h
**NG control**	135.7 ± 3.9	141.7 ± 4.1	127.5 ± 6.8	140.7 ± 11.2	122 ± 7.6
**STID + IELAc-0.5**	365.6 ± 15.6 ^*^	335.3 ±1 0.4 ^*,●^	316.3 ± 14.1 ^*,d^	368.3 ± 13.3 ^*^	412.6 ± 4.9 ^*^
**STID + IELAc-1.5**	342.3 ± 13.2 ^*^	276 ± 11.9 ^*,●,Ψ^	222.5 ± 4.2 ^*,●,Φ,Ψ^	227 ± 10.6 ^●,Ψ^	215.6 ± 12.9 ^*,●,Φ,Ψ^
**STID + IELAc-3.0**	348.0 ± 8.9 ^*^	296.6 ± 18.3 ^*,●^	222.6 ± 32.7 ^*,●,Φ,Ψ^	249.6 ± 38.3 ^*,●^	245.6 ± 33.4 ^*,●,Φ^
**STID + Aca**	371.0 ± 8.9 ^*^	301.0 ± 4.4 ^*,●,Ψ^	314.6 ± 12.0 ^*,Ψ^	320.0 ± 21.7 ^*^	376.0 ± 14.8 ^*^
**STID control**	368.3 ± 5.1 ^*^	391.6 ± 2.0 ^*^	379.0 ± 12.0 ^*^	354.6 ± 19.1 ^*^	386.0 ± 3.6 ^*^

Blood glucose level measurements of treated mice in the acute assay. The data are expressed as the mean ± SEM (*n* = 6); ^*^ *p* < 0.05 vs. normoglycemic NG control mice; ^●^ *p* < 0.05 vs. STID control mice; ^Φ^ *p* < 0.05 vs. standard drug control; ^Ψ^ *p* < 0.05 vs. initial value. NG, normoglycemic group; STID, streptozocin-induced diabetic control group; IELAc-0.5, group administered tea infusion extract from *A. cherimola* made with 0.5 g plant powder 300 mg/kg; IELAc-1.5, group administered tea infusion extract from *A. cherimola* made with 1.5 g plant powder 300 mg/kg; IELAc-3.0, group administered tea infusion extract from *A. cherimola* made with 3.0 g plant powder 300 mg/kg; Aca, group administered acarbose 50 mg/kg as pharmacological control.

**Table 3:** Effect of the tea infusion extract of leaves from *Annona cherimola* (IELAc-1.5) over blood glucose levels (mg/dL) on treated mice in subchronic assay.

	NG Mice		STID Mice		
	Control	IELAc-1.5	Control	IELAc-1.5	Aca
**Day 0**	115.2 ± 3.8 ^●^	128.6 ± 4.1 ^●,Φ^	333.2 ± 6.2 ^*^	324.5 ± 8.9 ^*^	324.5 ± 7.1 ^*^
**Day 7**	89.3 ± 2.7 ^●,Ψ^	115.2 ± 3.8 ^●,Φ,Ψ^	312.0 ± 11.3 ^*^	308 ± 17.6 ^*^	320.7 ± 3.9 ^*^
**Day 14**	128.0 ± 5.4 ^●^	142.0 ± 7.4 ^●,Φ^	343.2 ± 13.9 ^*^	327.0 ± 18.0 ^*^	366.0 ± 21.2 ^*^
**Day 21**	146.3 ± 3.7 ^●,Φ,Ψ^	154.3 ± 3.0 ^●,Φ^	375.0 ± 13.3	325.2 ± 5.7 ^*,●,Φ^	376.2 ± 13.9 ^*^
**Day 28**	122.0 ± 4.6 ^●^	145.3 ± 9.9 ^●,Φ^	398.5 ± 16.1 ^Ψ^	354.0 ± 16.5 ^*,Φ^	412.7 ± 15.5 ^*,Ψ^

Blood glucose level measurements (mg/dL) of all treated groups on the subchronic assay. The data are expressed as the mean ± SEM (*n* = 6). ^*^ *p* < 0.05 vs. NG control mice; ^●^ *p* < 0.05 vs. STID control mice; ^Φ^ *p* < 0.05 vs. standard drug control; ^Ψ^ *p* < 0.05 vs. initial value. NG, normoglycemic; IELAc-1.5, group administered tea infusion extract with 1.5 g of leaves from *A. cherimola* 300 mg/kg; Aca, group administered acarbose 50 mg/kg as pharmacological control.

**Table 4:** Effect of administration of the tea infusion extract of leaves from *Annona cherimola* on lipid profile and HbA1c.

	NG Mice		STID Mice		
	Vehicle	IELAc-1.5	Vehicle	IELAc-1.5	Aca
Blood glucose level (mg/dL)	120.1 ± 10.3	129 ± 12.8 ^●,Φ^	352.4 ± 17.1 ^*^	327.7 ± 8.2 ^*^	360.0 ± 19.1 ^*^
HbA1c (%)	6.3 ± 0.1	6.5 ± 0.1 ^Φ^	9.7 ± 0.5 ^*^	8.4 ± 0.4 ^●^	9.3 ± 0.5 ^*^
CHO (mg/dL)	96.0 ± 1.8	95.7 ± 1.7 ^●^	116.5 ± 3.8 ^*^	98.6 ± 2.0 ^●^	105.3 ± 1.8
TG (mg/dL)	141.9 ± 7.0	136.3 ± 7.3	144.9 ± 7.5	113.1 ± 9.8 ^*,●,Φ^	180.8 ± 6.9
HDL(mg/dL)	48.8 ± 2.7	52.3 ± 3.9	49.1 ± 3.8	59.8 ± 3.0 ^*^	52.6 ± 4.0
LDL (mg/dL)	17.8 ± 2.6	13.5 ± 4.6	28.0 ± 6.0	10.3 ± 3.2 ^●^	7.9 ± 1.8 ^●^

Values of lipid profile and glycated hemoglobin (HbA1c) of all treated groups. The data are expressed as the mean ± SEM (*n* = 6). ^*^ *p* < 0.05 vs. NG control mice; ^●^ *p* < 0.05 vs. STID control mice; ^Φ^ *p* < 0.05 vs. standard drug control. NG, normoglycemic; IELAc-1.5, group administered tea infusion extract with 1.5 g of leaves from *A. cherimola* 300 mg/kg; Aca, group administered acarbose 50 mg/kg as pharmacological control.

**Table 5:** The effect of IELAc-1.5 on body weight (g) of treated mice in subchronic study.

	NG Mice		STID Mice		
	Control (g)	IELA-1.5 (g)	Control (g)	IELAc-1.5 (g)	Aca (g)
**Day 0**	28.0 ± 0.57 ^●^	28.6 ± 0.88 ^●,Φ^	25.33 ± 0.57 ^*^	26.0 ± 0.57	26.0 ± 0.57
**Day 7**	28.3 ± 0.88 ^●^	29.6 ± 0.33 ^●,Φ^	24.33 ± 1.66 ^*^	25.3 ± 0.66 ^*^	24.0 ± 1.52 ^*^
**Day 14**	28.3 ± 0.88 ^●^	28.6 ± 0.66 ^●,Φ^	24.0 ± 1.15 ^*^	24.0 ± 1.15 ^*^	24.3 ± 2.18
**Day 21**	27.0 ± 0.57	28.6 ± 0.66 ^●,Φ^	23.3 ± 1.45	25.3 ± 1.45	24.0 ± 2.08
**Day 28**	27.3 ± 0.88 ^●^	28.6 ± 0.66 ^●,Φ^	22.6 ± 1.45 ^*^	24.3 ± 1.45	24.0 ± 1.05

Body weight (g) of all treated groups in the subchronic assay. The data are expressed as the mean ± SEM (*n* = 6). ^*^ *p* < 0.05 vs. NG control mice; ^●^ *p* < 0.05 vs. STID control mice; ^Φ^ *p* < 0.05 vs. standard drug control. NG, normoglycemic; IELAc-1.5, group administered tea infusion extract with 1.5 g of the leaves from *A. cherimola* 300 mg/kg; Aca, group administered acarbose 50 mg/kg as pharmacological control.

**Table 6:** Effect of the tea- infusion extract of leaves from *Annona cherimola* (IELAc-1.5) and acarbose (Aca) on urine profile in the subchronic assay.

Parameter	NG Mice		STID Mice		
	Vehicle	IELAc-1.5	Vehicle	IELAc-1.5	Aca
VOLUME (mL)	1.00 ± 0.346	0.83 ± 1.160	0.97 ± 0.179	0.76 ± 0.175	1.35 ± 0.221
BWC (Leu/μL)	NEG	NEG	NEG	NEG	NEG
NITRITE (mg/dL)	NEG	NEG	NEG	NEG	NEG
URO (mg/dL)	0.20	0.20	0.20	0.20	0.20
PROT (mg/dL)	30 ± 0.000	18.75 ± 3.750	22.5 ± 4.330	18.75 ± 3.750	18.75 ± 3.750
pH	6	6	6.125 ± 0.125	6	6.37 ± 0.375
BLOOD (mg/dL)	NEG	NEG	NEG	NEG	NEG
SG	1.02 ± 0.003	1.02 ± 0.004	1.01 ± 0.001	1.02 ± 0.001	1.02 ± 0.002
KET (mg/dL)	8.75 ± 3.750	6.25 ± 3.145	3.75 ± 1.250	2.50 ± 1.443	1.25 ± 1.250
BIL (mg/dL)	0.5 ± 0.288	0.25 ± 3.145	0.75 ± 0.250	0.50 ± 2.888	0.25 ± 0.250
GLU (mg/dL)	30.6 ± 15.324 ^●,Φ^	50.2 ± 28.860 ^●,Φ^	875 ± 125.311 ^*^	500 ± 223.606 ^*,●,Φ^	1000 ± 402.492 ^*^

Results of the urinalysis of the treated groups in the subchronic assay. The data are expressed as the mean ± SEM (*n* = 6). ^*^ *p* < 0.05 vs. NG control mice; ^●^ *p* < 0.05 vs. STID control mice; ^Φ^ *p* < 0.05 vs. standard drug control. NEG, negative; NG, normoglycemic; STID, streptozocin-induced diabetic mice; IELAc-1.5, group administered tea infusion extract with 1.5 g of leaves from *A. cherimola* 300 mg/kg; Aca, group administered acarbose 50 mg/kg as pharmacological control.

**Table 7:** Effects of the Tea Infusion Extract of Leaves from *Annona cherimola* (IELAc-1.5) on the Relative Weights (g) of Internal Organs on Treated Mice.

	NG Mice		STID Mice		
Organs	Vehicle (g)	IELAc-1.5 (g)	Vehicle (g)	IELAc-1.5 (g)	Aca (g)
Pancreas	0.81 ± 0.071	0.82 ± 0.037	0.71 ± 0.027 ^*^	0.87 ± 0.081	0.78 ± 0.060
Spleen	0.40 ± 0.030	0.46 ± 0.017	0.44 ± 0.038	0.53 ± 0.106	0.37 ± 0.023
Liver	4.71 ± 0.014 ^●^	5.16 ± 0.103 ^●,Φ^	5.87 ± 0.256 ^*^	6.15 ± 0.146 ^*^	6.35 ± 0.209 ^*^
Stomach	0.96 ± 0.06	1.06 ± 0.12	1.29 ± 0.16	1.39 ± 0.16 ^*^	1.36 ± 0.06
Gut	10.90 ± 0.760	9.76 ± 0.393 ^Φ^	11.63 ± 1.435	11.17 ± 0.816	13.08 ± 1.116
Kidneys	1.58 ± 0.037	1.69 ± 0.071	1.56 ± 0.027	1.55 ± 0.078	1.60 ± 0.041

Relative organ weight (ROW) (g) of the visceral organs of all treated groups in the subchronic assay. The data are expressed as the mean ± SEM (*n* = 6). ^*^ *p* < 0.05 vs. NG control mice; ^●^ *p* < 0.05 vs. STID control mice; ^Φ^ *p* < 0.05 vs. standard drug control. The index was calculated as the organ-to-body-weight ratio (relative weight, percentage). NG, normoglycemic; STID, streptozocin-induced diabetic mice; IELAc-1.5, group administered tea infusion extract with 1.5 g of leaves from *A. cherimola* 300 mg/kg; Aca, group administered acarbose 50 mg/kg.

## Data Availability

The data presented in this article are available on request from the corresponding author.

## Sample Availability

Samples used are available from the authors.

## References

[1] Guthrie R.A., Guthrie D.W. (2004). Pathophysiology of Diabetes Mellitus. Crit. Care Nurs. Q..

[2] DeFronzo R.A. (2009). From the Triumvirate to the Ominous Octet: A New Paradigm for the Treatment of Type 2 Diabetes Mellitus. Diabetes.

[3] Khursheed R., Singh S.K., Wadhwa S., Kapoor B., Gulati M., Kumar R., Ramanunny A.K., Awasthi A., Dua K. (2019). Treatment Strategies against Diabetes: Success so Far and Challenges Ahead. Eur. J. Pharmacol..

[4] (2019). IDF Diabetes Atlas 9th Edition. https://diabetesatlas.org/en/.

[5] García-Chapa E.G., Leal-Ugarte E., Peralta-Leal V., Durán-González J., Meza-Espinoza J.P. (2017). Genetic Epidemiology of Type 2 Diabetes in Mexican Mestizos. BioMed Res. Int..

[6] Association A.D. (2021). Pharmacologic Approaches to Glycemic Treatment: Standards of Medical Care in Diabetes—2021. Diabetes Care.

[7] Tan S.Y., Wong J.L.M., Sim Y.J., Wong S.S., Elhassan S.A.M., Tan S.H., Lim G.P.L., Rong Tay N.W., Annan N.C., Bhattamisra S.K. (2019). Type 1 and 2 Diabetes Mellitus: A Review on Current Treatment Approach and Gene Therapy as Potential Intervention. Diabetes Metab. Syndr..

[8] Shikov A.N., Pozharitskaya O.N., Makarov V.G. (2016). Aralia Elata Var. Mandshurica (Rupr. & Maxim.) J.Wen: An Overview of Pharmacological Studies. Phytomedicine.

[9] Yaseen G., Ahmad M., Zafar M., Sultana S., Kayani S., Cetto A.A., Shaheen S. (2015). Traditional Management of Diabetes in Pakistan: Ethnobotanical Investigation from Traditional Health Practitioners. J. Ethnopharmacol..

[10] Nowbandegani A.S., Kiumarcy S., Rahmani F., Dokouhaki M., Khademian S., Zarshenas M.M., Faridi P. (2015). Ethnopharmacological Knowledge of Shiraz and Fasa in Fars Region of Iran for Diabetes Mellitus. J. Ethnopharmacol..

[11] Shikov A.N., Pozharitskaya O.N., Makarova M.N., Kovaleva M.A., Laakso I., Dorman H.J.D., Hiltunen R., Makarov V.G., Galambosi B. (2012). Effect of Bergenia crassifolia L. Extracts on Weight Gain and Feeding Behavior of Rats with High-Caloric Diet-Induced Obesity. Phytomedicine.

[12] Madić V., Petrović A., Jušković M., Jugović D., Djordjević L., Stojanović G., Vasiljević P. (2021). Polyherbal Mixture Ameliorates Hyperglycemia, Hyperlipidemia and Histopathological Changes of Pancreas, Kidney and Liver in a Rat Model of Type 1 Diabetes. J. Ethnopharmacol..

[13] Chawla R., Thakur P., Chowdhry A., Jaiswal S., Sharma A., Goel R., Sharma J., Priyadarshi S.S., Kumar V., Sharma R.K. (2013). Evidence Based Herbal Drug Standardization Approach in Coping with Challenges of Holistic Management of Diabetes: A Dreadful Lifestyle Disorder of 21st Century. J. Diabetes Metab. Disord..

[14] Pineda-Ramírez N., Calzada F., Alquisiras-Burgos I., Medina-Campos O.N., Pedraza-Chaverri J., Ortiz-Plata A., Pinzón Estrada E., Torres I., Aguilera P. (2020). Antioxidant Properties and Protective Effects of Some Species of the Annonaceae, Lamiaceae, and Geraniaceae Families against Neuronal Damage Induced by Excitotoxicity and Cerebral Ischemia. Antioxidants.

[15] Andrade-Cetto A., Heinrich M. (2005). Mexican Plants with Hypoglycaemic Effect Used in the Treatment of Diabetes. J. Ethnopharmacol..

[16] Vega G., Esther M. (2013). Chirimoya (Annona Cherimola Miller), Frutal Tropical y Sub-Tropical de Valores Promisorios. Cultiv. Trop..

[17] Larranaga N., Albertazzi F.J., Fontecha G., Palmieri M., Rainer H., van Zonneveld M., Hormaza J.I. (2017). A Mesoamerican Origin of Cherimoya (Annona Cherimola Mill.): Implications for the Conservation of Plant Genetic Resources. Mol. Ecol..

[18] Calzada F., Correa-Basurto J., Barbosa E., Mendez-Luna D., Yepez-Mulia L. (2017). Antiprotozoal Constituents from Annona Cherimola Miller, a Plant Used in Mexican Traditional Medicine for the Treatment of Diarrhea and Dysentery. Pharmacogn. Mag..

[19] Quílez A.M., Fernández-Arche M.A., García-Giménez M.D., De la Puerta R. (2018). Potential Therapeutic Applications of the Genus Annona: Local and Traditional Uses and Pharmacology. J. Ethnopharmacol..

[20] Barbalho S.M., Goulart R., Farinazzi-Machado F.V., Souza M., Cincotto dos Santos Bueno P., Landgraf Guiguer E., Cressoni Araujo A., Groppo M. (2012). Annona Sp: Plants with Multiple Applications as Alternative Medicine—A Review. Curr. Bioact. Compd..

[21] Leite D.O.D., de F A Nonato C., Camilo C.J., de Carvalho N.K.G., da Nobrega M.G.L.A., Pereira R.C., da Costa J.G.M. (2020). Annona Genus: Traditional Uses, Phytochemistry and Biological Activities. Curr. Pharm. Des..

[22] Díaz-de-Cerio E., Aguilera-Saez L.M., Gómez-Caravaca A.M., Verardo V., Fernández-Gutiérrez A., Fernández I., Arráez-Román D. (2018). Characterization of Bioactive Compounds of Annona Cherimola L. Leaves Using a Combined Approach Based on HPLC-ESI-TOF-MS and NMR. Anal. Bioanal. Chem..

[23] Albuquerque T.G., Santos F., Sanches-Silva A., Oliveira M., Bento A., Costa H. (2014). Nutritional and Phytochemical Composition of Annona Cherimola Mill. Fruits and by-Products: Potential Health Benefits. Food Chem..

[24] Mannino G., Gentile C., Porcu A., Agliassa C., Caradonna F., Bertea C.M. (2020). Chemical Profile and Biological Activity of Cherimoya (Annona Cherimola Mill.) and Atemoya (Annona Atemoya) Leaves. Molecules.

[25] Martínez-Vázquez M., Estrada-Reyes R., Escalona A.A., Velázquez I.L., Martínez-Mota L., Moreno J., Heinze G. (2012). Antidepressant-like Effects of an Alkaloid Extract of the Aerial Parts of Annona Cherimolia in Mice. J. Ethnopharmacol..

[26] Ammoury C., Younes M., El Khoury M., Hodroj M.H., Haykal T., Nasr P., Sily M., Taleb R.I., Sarkis R., Khalife R. (2019). The Pro-Apoptotic Effect of a Terpene-Rich Annona Cherimola Leaf Extract on Leukemic Cell Lines. BMC Complement. Altern. Med..

[27] OECD/OCDE (2001). Guideline for Testing of Chemicals. Acute Oral Toxicity-Acute Toxic Class Method. https://ntp.niehs.nih.gov/Iccvam/suppdocs/feddocs/oecd/oecd_gl423.pdf.

[28] OECD Test Guidelines for Chemicals-OECD. http://www.oecd.org/chemicalsafety/testing/oecdguidelinesforthetestingofchemicals.htm.

[29] El-Tantawy W.H., Temraz A. (2018). Management of Diabetes Using Herbal Extracts: Review. Arch. Physiol. Biochem..

[30] Giovannini P., Howes M.-J.R., Edwards S.E. (2016). Medicinal Plants Used in the Traditional Management of Diabetes and Its Sequelae in Central America: A Review. J. Ethnopharmacol..

[31] Choudhury H., Pandey M., Hua C.K., Mun C.S., Jing J.K., Kong L., Ern L.Y., Ashraf N.A., Kit S.W., Yee T.S. (2018). An Update on Natural Compounds in the Remedy of Diabetes Mellitus: A Systematic Review. J. Tradit. Complement. Med..

[32] Peesa J.P. (2013). Herbal Medicine for Diabetes Mellitus: A Review. Int. J. Phytopharm..

[33] Matheka D., Alkizim F. (2012). Complementary and Alternative Medicine for Type 2 Diabetes Mellitus: Role of Medicinal Herbs. J. Diabetes Endocrinol..

[34] Florence N.T., Benoit M.Z., Jonas K., Alexandra T., Désiré D.D.P., Pierre K., Théophile D. (2014). Antidiabetic and Antioxidant Effects of Annona Muricata (Annonaceae), Aqueous Extract on Streptozotocin-Induced Diabetic Rats. J. Ethnopharmacol..

[35] Justino A.B., Miranda N.C., Franco R.R., Martins M.M., da Silva N.M., Espindola F.S. (2018). Annona Muricata Linn. Leaf as a Source of Antioxidant Compounds with in Vitro Antidiabetic and Inhibitory Potential against α-Amylase, α-Glucosidase, Lipase, Non-Enzymatic Glycation and Lipid Peroxidation. Biomed. Pharmacother..

[36] Adewole S.O., Ojewole J.A.O. (2008). Protective Effects of Annona Muricata Linn. (Annonaceae) Leaf Aqueous Extract on Serum Lipid Profiles and Oxidative Stress in Hepatocytes of Streptozotocin-Treated Diabetic Rats. Afr. J. Tradit. Complement. Altern. Med. AJTCAM.

[37] Kaleem M., Asif M., Ahmed Q.U., Bano B. (2006). Antidiabetic and Antioxidant Activity of Annona Squamosa Extract in Streptozotocin-Induced Diabetic Rats. Singap. Med. J..

[38] Kaur R., Afzal M., Kazmi I., Ahamd I., Ahmed Z., Ali B., Ahmad S., Anwar F. (2013). Polypharmacy (Herbal and Synthetic Drug Combination): A Novel Approach in the Treatment of Type-2 Diabetes and Its Complications in Rats. J. Nat. Med..

[39] Victoria Amador M.D.C., Morón Rodríguez F., Morejón Rodríguez Z., Martínez Guerra M.J., López Barreiro M. (2006). Tamizaje Fitoquímico, Actividad Antiinflamatoria y Toxicidad Aguda de Extractos de Hojas de Annona Squamosa L.. Rev. Cuba. Plantas Med..

[40] Taderera T., Chagonda L., Gomo E., Katerere D., Shai L.J. (2019). Annona Stenophylla Aqueous Extract Stimulate Glucose Uptake in Established C2Cl2 Muscle Cell Lines. Afr. Health Sci..

[41] Brindis F., González-Trujano M.E., González-Andrade M., Aguirre-Hernández E., Villalobos-Molina R. (2013). Aqueous Extract of Annona Macroprophyllata: A Potential α-Glucosidase Inhibitor. BioMed Res. Int..

[42] Carballo A., Martínez A., González-Trujano M., Pellicer F., Ventura-Martinez R., Diazreval I., López-Muñoz F. (2009). Antinociceptive Activity of Annona Diversifolia Saff. Leaf Extracts and Palmitone as a Boactive Compound. Pharmacol. Biochem. Behav..

[43] Valdés M., Calzada F., Mendieta-Wejebe J.E., Merlín-Lucas V., Velázquez C., Barbosa E. (2020). Antihyperglycemic Effects of Annona Diversifolia Safford and Its Acyclic Terpenoids: α-Glucosidase and Selective SGLT1 Inhibitiors. Molecules.

[44] Verma A.M., Kumar A.P., Shekar R.K., Kumar K.A. (2011). Pharmacological Screening of *Annona cherimola* for Antihyperlipidemic Potential. J. Basic Clin. Pharm..

[45] Falé P.L., Ferreira C., Maruzzella F., Florêncio M.H., Frazão F.N., Serralheiro M.L. (2013). Evaluation of Cholesterol Absorption and Biosynthesis by Decoctions of Annona Cherimola Leaves. J. Ethnopharmacol..

[46] Santos S.A.O., Vilela C., Camacho J.F., Cordeiro N., Gouveia M., Freire C.S.R., Silvestre A.J.D. (2016). Profiling of Lipophilic and Phenolic Phytochemicals of Four Cultivars from Cherimoya (Annona Cherimola Mill.). Food Chem..

[47] Arthur F.K..N., Woode E., Terlabi E.O., Larbie C. (2011). Evaluation of Acute and Subchronic Toxicity of *Annona Muricata* (Linn.) Aqueous Extract in Animals. Eur. J. Exp. Biol..

[48] Sanchez-Gonzales G., Castro-Rumiche C., Alvarez-Guzman G., Flores-García J., Barriga-Sánchez M., Sanchez-Gonzales G., Castro-Rumiche C., Alvarez-Guzman G., Flores-García J., Barriga-Sánchez M. (2019). Phenolic Compounds and Antioxidant Activity of Extracts from Chirimoya (Annona Cherimola Mill) Leaf. Rev. Colomb. Quím..

[49] Galarce-Bustos O., Fernández-Ponce M.T., Montes A., Pereyra C., Casas L., Mantell C., Aranda M. (2020). Usage of Supercritical Fluid Techniques to Obtain Bioactive Alkaloid-Rich Extracts from Cherimoya Peel and Leaves: Extract Profiles and Their Correlation with Antioxidant Properties and Acetylcholinesterase and α-Glucosidase Inhibitory Activities. Food Funct..

[50] Calzada F., Solares-Pascasio J.I., Ordoñez-Razo R.M., Velazquez C., Barbosa E., García-Hernández N., Mendez-Luna D., Correa-Basurto J. (2017). Antihyperglycemic Activity of the Leaves from Annona Cherimola Miller and Rutin on Alloxan-Induced Diabetic Rats. Pharmacogn. Res..

[51] Maritim A.C., Sanders R.A., Watkins J.B. (2003). Diabetes, Oxidative Stress, and Antioxidants: A Review. J. Biochem. Mol. Toxicol..

[52] Gan T., Liao B., Xu G. (2018). The Clinical Usefulness of Glycated Albumin in Patients with Diabetes and Chronic Kidney Disease: Progress and Challenges. J. Diabetes Complicat..

[53] Kovatchev B.P. (2017). Metrics for Glycaemic Control - from HbA1c to Continuous Glucose Monitoring. Nat. Rev. Endocrinol..

[54] Asgharpour F., Pouramir M., Khalilpour A., Alamdar S.A., Rezaei M. (2013). Antioxidant Activity and Glucose Diffusion Relationship of Traditional Medicinal Antihyperglycemic Plant Extracts. Int. J. Mol. Cell. Med..

[55] Tadera K., Minami Y., Takamatsu K., Matsuoka T. (2006). Inhibition of α-Glucosidase and α-Amylase by Flavonoids. J. Nutr. Sci. Vitaminol..

[56] Jo S.-H., Ka E.-H., Lee H.-S., Apostolidis E., Jang H.-D., Kwon Y.-I. (2009). Comparison of Antioxidant Potential and Rat Intestinal A-Glucosidases Inhibitory Activities of Quercetin, Rutin, and Isoquercetin.

[57] Yang J., Guo J., Yuan J. (2008). In Vitro Antioxidant Properties of Rutin. LWT-Food Sci. Technol..

[58] Sharma S., Ali A., Ali J., Sahni J.K., Baboota S. (2013). Rutin: Therapeutic Potential and Recent Advances in Drug Delivery. Expert Opin. Investig. Drugs.

[59] Ghorbani A. (2017). Mechanisms of Antidiabetic Effects of Flavonoid Rutin. Biomed. Pharmacother..

[60] Kamalakkannan N., Prince P.S.M. (2006). Antihyperglycaemic and Antioxidant Effect of Rutin, a Polyphenolic Flavonoid, in Streptozotocin-Induced Diabetic Wistar Rats. Basic Clin. Pharmacol. Toxicol..

[61] Alam F., Shafique Z., Amjad S.T., Bin Asad M.H.H. (2019). Enzymes Inhibitors from Natural Sources with Antidiabetic Activity: A Review. Phytother. Res..

[62] Solares-Pascasio J.I., Ceballos G., Calzada F., Barbosa E., Velazquez C. (2021). Antihyperglycemic and Lipid Profile Effects of *Salvia amarissima* Ortega on Streptozocin-Induced Type 2 Diabetic Mice. Molecules.

[63] Kim J.H., Kang M.J., Choi H.N., Jeong S.M., Lee Y.M., Kim J.I. (2011). Quercetin attenuates fasting and postprandial hyperglycemia in animal models of diabetes mellitus. Nutr. Res. Pract..

[64] Fischer S., Hanefeld M., Spengler M., Boehme K., Temelkova-Kurktschiev T. (1998). European study on dose-response relationship of acarbose as a first-line drug in non-insulin-dependent diabetes mellitus: Efficacy and safety of low and high doses. Acta Diabetol..

[65] Strain W.D., Paldánius P.M. (2018). Diabetes, Cardiovascular Disease and the Microcirculation. Cardiovasc. Diabetol..

[66] Wong N.K.P., Nicholls S.J., Tan J.T.M., Bursill C.A. (2018). The Role of High-Density Lipoproteins in Diabetes and Its Vascular Complications. Int. J. Mol. Sci..

[67] Srivastava R.A.K. (2018). Dysfunctional HDL in Diabetes Mellitus and Its Role in the Pathogenesis of Cardiovascular Disease. Mol. Cell. Biochem..

[68] Schwartz S.L. (2006). Diabetes and Dyslipidaemia. Diabetes Obes. Metab..

[69] Alvarez C.A., Lingvay I., Vuylsteke V., Koffarnus R.L., McGuire D.K. (2015). Cardiovascular Risk in Diabetes Mellitus: Complication of the Disease or of Antihyperglycemic Medications. Clin. Pharmacol. Ther..

[70] Wen W., Lin Y., Ti Z. (2019). Antidiabetic, Antihyperlipidemic, Antioxidant, Anti-Inflammatory Activities of Ethanolic Seed Extract of *Annona reticulata* L. in Streptozotocin Induced Diabetic Rats. Front. Endocrinol..

[71] Gothai S., Ganesan P., Park S.-Y., Fakurazi S., Choi D.-K., Arulselvan P. (2016). Natural Phyto-Bioactive Compounds for the Treatment of Type 2 Diabetes: Inflammation as a Target. Nutrients.

[72] Jung U.J., Lee M.-K., Park Y.B., Kang M.A., Choi M.-S. (2006). Effect of Citrus Flavonoids on Lipid Metabolism and Glucose-Regulating Enzyme MRNA Levels in Type-2 Diabetic Mice. Int. J. Biochem. Cell Biol..

[73] Vasarri M., Barletta E., Vinci S., Ramazzotti M., Francesconi A., Manetti F., Degl’Innocenti D. (2020). Annona Cherimola Miller Fruit as a Promising Candidate against Diabetic Complications: An In Vitro Study and Preliminary Clinical Results. Foods.

[74] Corcoran M., McKay D., Blumberg J. (2012). Flavonoid Basics: Chemistry, Sources, Mechanisms of Action, and Safety. J. Nutr. Gerontol. Geriatr..

[75] Diario Oficial de la Federación (1999). NOM-062-ZOO-1999, 2001. Norma Oficial Mexicana. Especificaciones Técnicas Para La Producción, Cuidado y Uso de Los Animales de Laboratorio. Diario Oficial de La Federación. 6 de Diciembre de 1999.

[76] Ngueguim F., Théophile D., Dzeufiet P., Bertin V., Etienne D., Beauwens R., Acha A., Louis Z., Kamtchouing P. (2007). Antidiabetic Activities of Methanol-Derived Extract of Dorstenia picta Twigs in Normal and Streptozotocin-Induced Diabetic Rats. Asian J. Tradit. Med..

[77] Calzada F., Valdes M., Garcia-Hernandez N., Velázquez C., Barbosa E., Bustos-Brito C., Quijano L., Pina-Jimenez E., Mendieta-Wejebe J.E. (2019). Antihyperglycemic activity of the leaves from *Annona diversifolia* Safford. and farnesol on normal and alloxan-induced diabetic mice. Pharmacogn Mag..

[78] OECD/OCDE (2008). Guideline for Testing of Chemicals. Repeated Dose 28-Day Oral Toxicity Study in Rodents. https://ntp.niehs.nih.gov/iccvam/suppdocs/feddocs/oecd/oecdtg407-2008.pdf.

[79] Maroo J., Vasu V.T., Aalinkeel R., Gupta S. (2002). Glucose Lowering Effect of Aqueous Extract of *Enicostemma littorale* Blume in Diabetes: A Possible Mechanism of Action. J. Ethnopharmacol..

[80] Friedewald W.T., Levy R.I., Fredrickson D.S. (1972). Estimation of the Concentration of Low-Density Lipoprotein Cholesterol in Plasma, Without Use of the Preparative Ultracentrifuge. Clin. Chem..

